# Ultra-thin 2D bimetallic MOF nanosheets for highly sensitive and stable detection of glucose in sweat for dancer

**DOI:** 10.1186/s11671-023-03838-0

**Published:** 2023-04-08

**Authors:** Yufei Mao, Tangchun Chen, Yifan Hu, KwanJung Son

**Affiliations:** 1 Department of Dance, Hanyang University, Seoul, 04763 Korea; 2 Department of Dance, Sichuan Conservatory of Music, Chengdu, 610500 China; 3 Department of Music, Changshu Institute of Technology, Changshu, 215500 China

**Keywords:** MOFs, Carbon cloth, Flexible biosensor, Noninvasive glucose monitoring

## Abstract

The measurement of glucose concentration in sweat is expected to replace the existing blood glucose detection, which realize the effective way of non-invasive monitoring of human glucose concentration in dancing. High precision glucose detection can be achieved by adjusting the electrode material of the sensor. Thus, in this work, the bimetallic organic frameworks (bi-MOFs) materials containing Mn and Ni ions (NiMn-MOF) with ultrathin nanosheets have been exquisitely designed. The ultrathin nanosheet and heterogeneous metal ions in the structure optimize the electronic structure, which improves the electrical conductivity of MOFs. The success of the preparation strategy leads the good electrocatalytic performance of NiMn-MOF for glucose detection. Detailedly, NiMn-MOF shows high sensitivity of 1576 μA mM^−1^ cm^−2^ in the linear range from 0 to 0.205 mM and the wide linear region of 0.255–2.655 mM and 3.655–5.655 mM were also observed. In addition, the high repeatability, reproductivity, long-term stability and ultra-low limited of detection (LOD, 0.28 μM, *S*/*N* = 3) provide foundation for the practical sensor application of this NiMn-MOF nanosheets. Remarkably, as designed NiMn-MOF sensor can accurately measure glucose in sweat showing great potential in the field of wearable glucose monitoring during dancing.

## Introduction

Glucose is an important nutrient for human cells. The concentration of glucose is an important parameter for the diagnosis of endocrine, metabolic disorders and diabetes [1, 2]. The glucose concentration in human body must be maintained within the normal range, too high or too low will cause great impact on human health and even threaten life. Therefore, the development of sensors with high sensitivity and stability for glucose detecting is particularly important for human health management [3]. Currently, diabetics need to take several measurements every day to monitor their blood sugar levels to ensure the correct dosage of medication, which requires that the patient can do it alone. Furthermore, the glucose content of human blood is tested by collecting blood at the fingertips and frequent pricking finger causes great pain and risk of infection. Furthermore, the test can not monitor the amount of glucose in the body during dancing, which is crucial for monitor the physical quality of dancers during dancing. It is well known that glucose is also found in human sweat and its concentration can change with the physiological conditions of the body, which can be monitored by electrochemical sensing technology [4]. Wearable sweat biosensors offer advanced means to effectively detect the state of human biomolecules and show great potential for monitoring of personal health and fitness [5, 6]. However, the content of glucose in sweat is relatively small, which puts forward higher requirements for the sensitivity and detection limit of glucose sensor. The electrode material is the main factor that determines the performance of electrochemical glucose sensor [7, 8]. Therefore, by modifying the electrode material with appropriate device structure, a high performance sensor for glucose monitoring in dance state can be obtained.

Metal–organic frameworks (MOFs) have become a new type of glucose sensitive material due to the high surface area, adjustable pore structure and diversified organic ligands and metal ions [9, 10]. However, the poor electrical conductivity severely hinders the glucose catalytic performance of MOFs [11]. Previous reports have shown that the electrical conductivity of MOFs can be improved by regulating the electronic structure of MOFs. The electronic structure of MOFs is greatly influenced by the topology and coordination environment. For example, missing ligands, introduction of heterogeneous metal ions and substitution of ligands in MOFs were shown to be effective in tuning the electronic structure of MOFs [12]. In addition, 2D MOFs nanomaterials are also good choices for high performance glucose sensitive materials due to their unique structure and abundant unsaturated coordination metal active sites [13].

In this work, the ultrathin nanosheet bimetallic MOFs were successfully fabricated by a simple one-step hydrothermal process. The three-dimensional nanoflower structure composed of ultrathin nanosheets not only reduces the path of ion diffusion in catalytic process but also exposes a large number of active sites to participate in catalytic reactions, which makes as prepared NiMn-MOF possessed remarkable sensing performance for glucose detection. Additionally, the sensor fabricated with the designed bimetallic material showed a significant response to glucose in sweat, demonstrating the potential of NiMn-MOF for wearable detection of glucose during the dancing process.

## Experiment section

### Preparation of NiMn-MOF

All chemicals were used directly without treatment. The NiMn-MOF was fabricated by a simple and designed synthesis strategy. Firstly, 2 mmol Ni(NO_3_)_2_·6H_2_O, 1 mmol MnCl_2_·4H_2_O and 2 mmol 1,3,5-Benzenetricarboxylic acid (H_3_BTC) was absolutely dissolved in 60 mL ethanol. After stirring for 1 h, the mixture was carefully transferred to an autoclave, reacted 10 h at 150 ℃. After cooling down to room temperature, sediment was collected by centrifugal cleaned by ethanol and dried. The product as prepared is NiMn-MOF.

### Fabrication of NiMn-MOF electrode and sensor

**Fig. 1:**
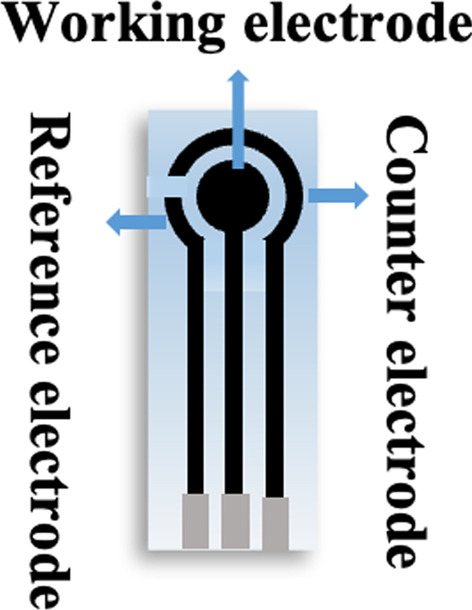
The structure of flexible wearable biosensor

The work electrode of as prepared NiMn-MOF on the glassy carbon electrodes (GCEs, $\emptyset = 3 \;{\text{mm}}$). Briefly, the GCEs were cleaned by the different sizes of polishing power. Then, 5 mg NiMn-MOF, 100 μL Nafion solution and 900 μL ultrapure water were mixed by the ultrasonic method. 5 μL of NiMn-MOF drops were added to the surface of GCEs to form ultra-thin film sensitive electrode at room temperature. The flexible wearable NiMn-MOF detector was fabricated by dripping the mixture on the surface of the working electrode as shown in Fig. 1.

### Material characterizations

The surface morphology was ascertained by the field-emission scanning electron microscope (FESEM, Ziess Gemini 300). The thickness of the NiMn-MOF was analyzed by the atomic force microscopy (AFM, Bruker Dimension Icon). X-ray photoelectron spectroscopy (XPS, Thermo Scientific K-Alpha) was utilized to analyze the surface constituents and valence states.

### Electrochemical measurement

The electro-chemical workstation (CHI660D, Chenhua, Shanghai, China) was employed to analysis the electrochemical performance of as designed NiMn-MOF. The tested solution is 0. M NaOH. The modified GCEs, platinum disk and Ag/AgCl were introduced as the working electrode, counter electrode and reference electrode, respectively. The electrochemical behavior of NiMn-MOF was tested by the cyclic voltammetry (CV). The chronoamperometry (CA) was utilized to determine the sensing property of as prepared products. The practical application of as designed biosensor was recorded by the BIOSYS intelligent biosensing system (Refresh Intelligent Technology Co. Ltd.).

## Results and discussion

### Morphology of NiMn-MOF

**Fig. 2:**
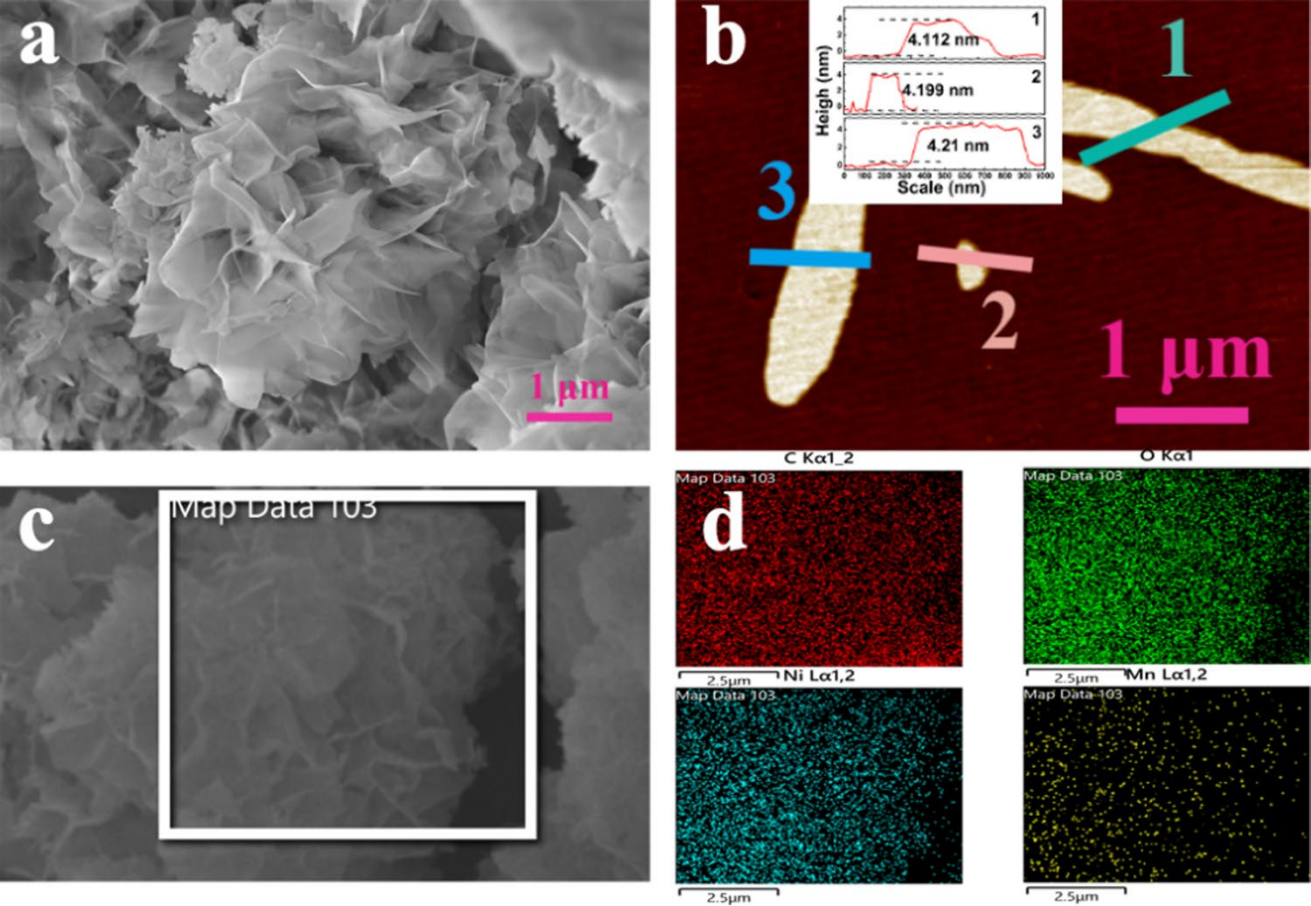
**a** The SEM image of NiMn-MOF. **b** The AFM spectrum of NiMn-MOF. **c** The HAADF image of NiMn-MOF. **d** The distribution diagram of element NiMn-MOF nanosheets

Figure 2a shows the SEM image of as prepared NiMn-MOF. It can be clearly seen that, the NiMn-MOF presents a three-dimensional nanoflower structure composed of two-dimensional nanosheets. The two-dimensional nanosheets are connected to each other with a honeycomb like network structure. The porous honeycomb structure would provide a quick transfer channel for ion transport during glucose oxidation. In addition, the ultra-thin nanosheets are highly opened, which reduces agglomeration and provides more active sites for catalytic reactions. Figure 2b shows the AFM spectrum of NiMn-MOF. Remarkably, the average thickness of as prepared NiMn-MOF is 4.17 nm. The ultrathin thickness further makes sure the complete exposure of active sites in the bimetallic material. Additionally, the ultra-thin nanosheet enables the ions of the electrolyte to better enter the material and improve the activity of the reaction, which will greatly enhance the oxidation performance of NiMn-MOF to glucose. Figure 2c and d shows the HAADF image and the corresponding elements distribution images of NiMn-MOF. It can be clearly seen that, the C, O, Ni and Mn elements are evenly distributed in the NiMn-MOF.

**Fig. 3:**
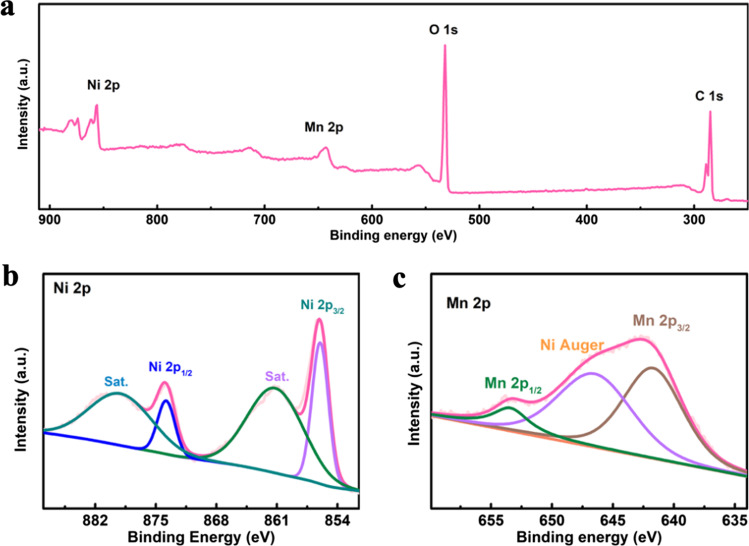
**a** The full XPS spectrum of NiMn-MOF. **b** Ni 2*p*. **c** Mn 2*p*

The surface components and valence states of NiMn-MOF were analyzed by XPS characterization. The full spectrum of Fig. 3a confirmed the existence of Ni, Mn, C, O elements. In the Ni 2*p* spectrum of NiMn-MOF (Fig. 3b), the fitted peaks at 856.0 eV and 873.8 eV are ascribed to Ni^2+^ 2*p*_3/2_ and Ni^2+^ 2*p*_1/2_, respectively. The two peaks at 861.2 eV and 879.1 eV are attributed to the satellite peak of Ni 2*p*_3/2_ and Ni 2*p*_1/2_, respectively. Figure 3c displays the Mn 2*p* high-resolution spectrum of NiMn-MOF. The Gaussian fitted peaks at 641.7 eV, 646.5 eV and 653.4 eV belong to the Mn 2*p*_3/2_, Ni Auger and Mn 2*p*_1/2_ of Mn^2+^, respectively.

### Electrochemical performance of NiMn-MOF

**Fig. 4:**
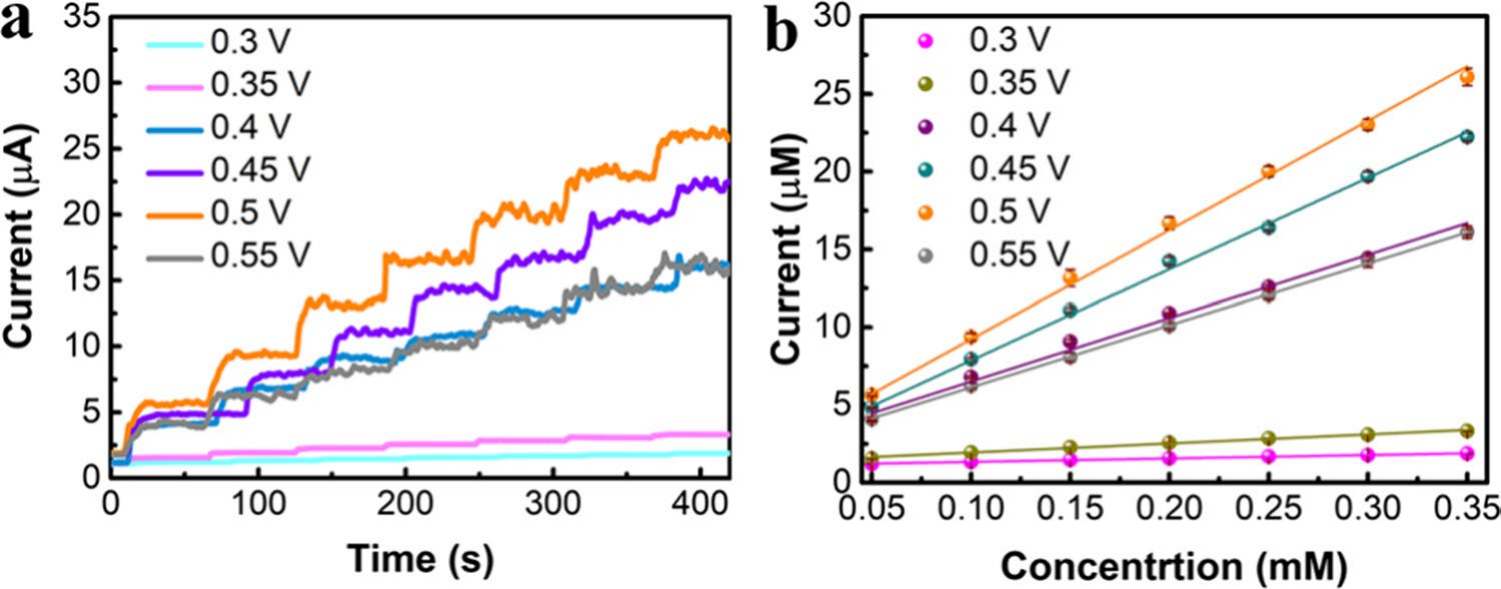
**a** The *i*–*t* curves of NiMn-MOF with various working voltage. **b** Corresponding fitting curves of (**a**)

The operating voltage can affect the catalytic performance of the electrode for glucose detection. Figure 4a shows the current response curves of NiMn-MOF with different applied potential in the 50 μM glucose. As shown in Fig. 3b, the *i*–*t* current response signal of NiMn-MOF in 0.3 and 0.4 V is smaller. Then, the current response increases with the operating voltage until the potential is set to 0.5 V. The oxidation current of NiMn-MOF to glucose is maximum under the 0.5 V, which can be verified by the maximum slope of 0.5 V (Fig. 4b). Thus, the 0.5 V is applied as the operating voltage in subsequent measurement. The detection mechanism of NiMn-MOF to glucose can be formulated as Eqs. (1)–(4) [14]. Firstly, the metal ions ${\text{Ni}}( {{\text{II}}})$ and ${\text{Mn}}( {{\text{II}}})$ in the bimetallic MOF react with the alkaline ions adsorbed on the electrode surface to form the high-valence metal ions. Then, metal ions in the high oxidation state oxidize glucose molecules to form gluconolactone.

1 $${\text{Ni}}({\text{II}}) - {\text{MOF}} + {\text{OH}}_{{{\text{sol}}}} ^{ - } \rightleftharpoons ~{\text{Ni}}({\text{III}}) - ({\text{OH}}){\text{MOF}}+ e^{ - }$$

2 $${\text{Mn}}({\text{II}}) - {\text{MOF}} + {\text{OH}}_{{{\text{sol}}}} ^{ - } ~ \rightleftharpoons ~{\text{Mn}}({\text{III}}) - ({\text{OH}}){\text{MOF}} + e^{ - }$$

3 $$2{\text{Ni}}({\text{III}}) - ({\text{OH}}){\text{MOF}} + {\text{C}}_{6} {\text{H}}_{{12}} {\text{O}}_{6} \rightleftharpoons 2{\text{Ni}}({\text{II}}) - {\text{MOF}} + {\text{C}}_{6} {\text{H}}_{{10}} {\text{O}}_{6} + 2{\text{H}}_{2} {\text{O}}$$

4 $$2{\text{Mn}}({\text{III}}) - ({\text{OH}}){\text{MOF}} + {\text{C}}_{6} {\text{H}}_{{12}} {\text{O}}_{6} \rightleftharpoons 2{\text{Mn}}({\text{II}}) - {\text{MOF}} + {\text{C}}_{6} {\text{H}}_{{10}} {\text{O}}_{6} + 2{\text{H}}_{2} {\text{O}}.$$

### Detection of glucose

**Fig. 5:**
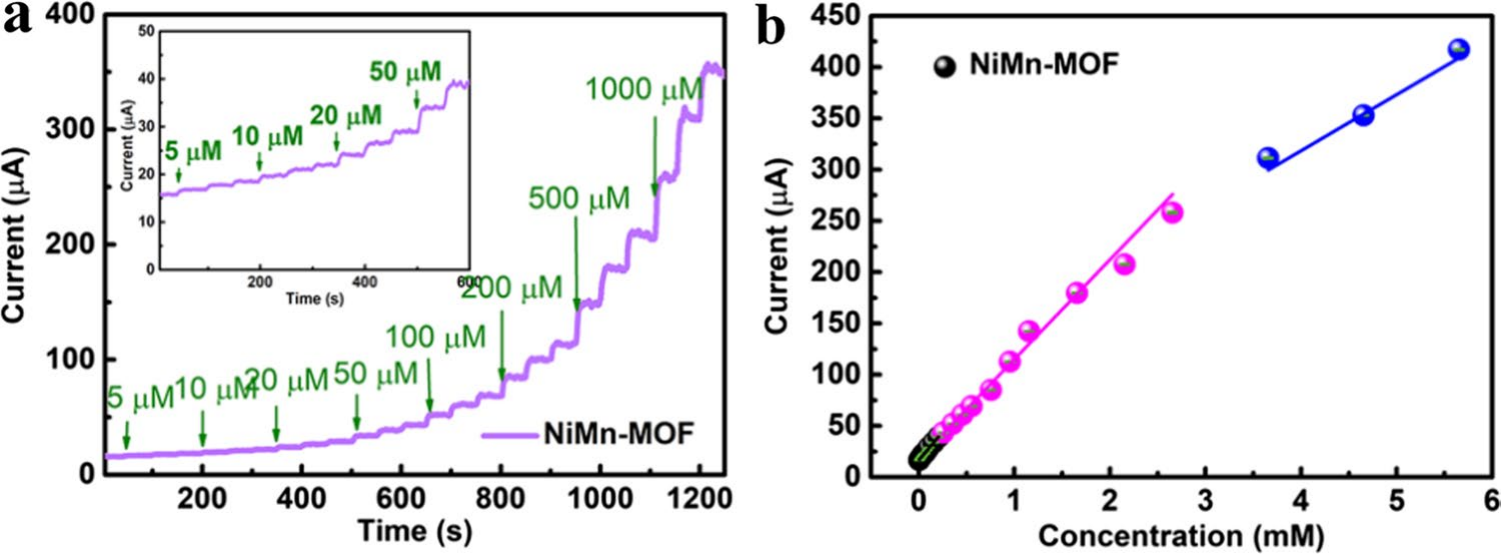
**a** The *i*–*t* curve of NiMn-MOF with different concentration of glucose. **b** Corresponding fitting curves of (**a**)

The catalytic property of NiMn-MOF to glucose was investigated by the CA method. Figure 5a is the current response of NiMn-MOF to glucose with various concentration. The dropped concentration of glucose is from 5 to 1000 μM. The stepwise ascending *i*–*t* curve shows that as designed NiMn-MOF shows obvious electrocatalytic properties with glucose. It is worth noting that, the response current increased with the increase of glucose concentration. In addition, the steady increase of *i*–*t* signal indicates that NiMn-MOF has a good stability in the detection process of glucose. Figure 5b shows the corresponding fitting curves between glucose concentration and response current. Three linear ranges of 0–0.205 mM, 0.255–2.655 mM and 3.655–5.655 mM are observed in the fitted curves. According to the slopes of fitted curves in Fig. 4b, the sensitivity of NiMn-MOF in the linear range are 1576 (0–0.205 mM), 1285 (0.255–2.655 mM) and 755.7 μA mM^−1^ cm^−2^ (3.655–5.655 mM), respectively. In addition, the low limitation of detection about 0.28 μM (*S*/*N* = 3) was acquired. The performance comparison of NiMn-MOF and other MOF-based electrochemical glucose sensors is shown in Table 1 [15]. As shown in the table, compared with other materials, NiMn-MOF nanosheet designed in this study has high sensitivity and low detection limit. The excellent catalytic performance of NiMn-MOF can be attributed to the ultrathin nanosheet structure and the introduction of heterogeneous metal ions, which can strongly facilitate exposure of the active site, providing a large number of reactive sites for glucose oxidation.

**Table 1:** Comparison of previous reported glucose sensing based on MOFs

Materials	Linear ranges (μM)	Detection limit (μM)	Sensitivity (μA mM^−1^ cm^−2^)	References
Au/MOF (Fe, Mn)/CNTs^a^	0.005–0.3	0.002	–	[15]
Ag/ZIF-67/GCE^b^	2–1000	0.66	379	[16]
Co-MOF/EG^c^	1–3330	0.58	330	[17]
ZIF-67NiCo NSs^d^/GNR^e^	5–800	0.6	344	[18]
Ni-MOF/RGO/CF	6–2090	–	852	[19]
Ni-MOF/NF	40–2000	0.085	–	[20]
N–Co-MOF	1–2000	0.5	–	[10]
Ni–Co MOF/Au/PDMS	20–790	–	205.1	[6]
Ni–Co MOF/Ag/rGO/PU	10–660	–	425.9	[8]
NiMn-MOF	5–5655	0.28	1576	This work

^a^CNT: carbon nanotubes

^b^GCE: glassy carbon electrode

^c^EG: exfoliated graphene

^d^NSs: nanosheets

^e^GNR: graphene nanoribbons

### Reproductivity, repeatability and stability of NiMn-MOF electrode

**Fig. 6:**
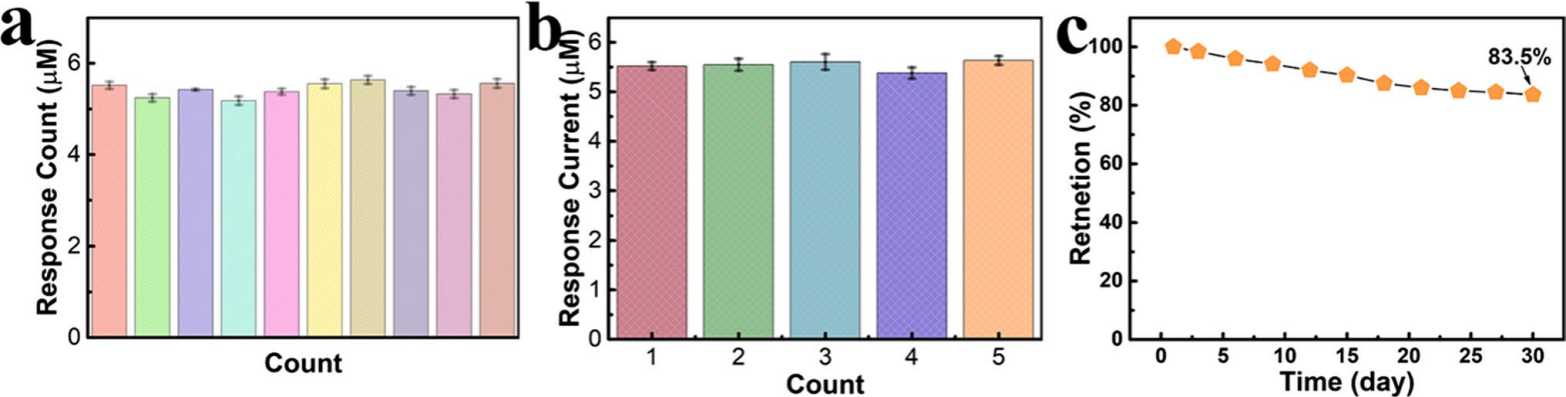
**a** The response current of NiMn-MOF in ten times measurement. **b** The *i*–*t* signal of five electrodes NiMn-MOF with the same fabricated process. **c** The long-term stability of NiMn-MOF

The repeatability of the NiMn-MOF electrode was investigated by testing the same electrode by ten times. As shown in Fig. 6a, the relative standard deviation (RSD) of these ten measurements is less than 2.7%. The low RSD of ten measurements from the same electrode indicates that the designed NiMn-MOF has good reversibility, which possesses great potential in the field of reusable electrode materials for glucose sensors. The reproducibility is also an important parameter in the fabrication of electrochemical biosensor electrode materials. The reproducibility of NiMn-MOF electrode was analyzed by testing 5 different electrodes prepared from different batches (Fig. 6b). The remarkable reproducibility of NiMn-MOF is evidenced by the ultra-low RSD of 1.8%. In addition, the long-term stability of the prepared NiMn-MOF electrode was tested within 30 days. As shown in Fig. 6c, the electrode still maintains 83.5% of the original response after 1 month testing. The excellent repeatability, reproductivity and long-term stability of NiMn-MOF electrode are strongly conducive to improving the practical applications of as designed wearable devices.

### Real sample detection

**Fig. 7:**
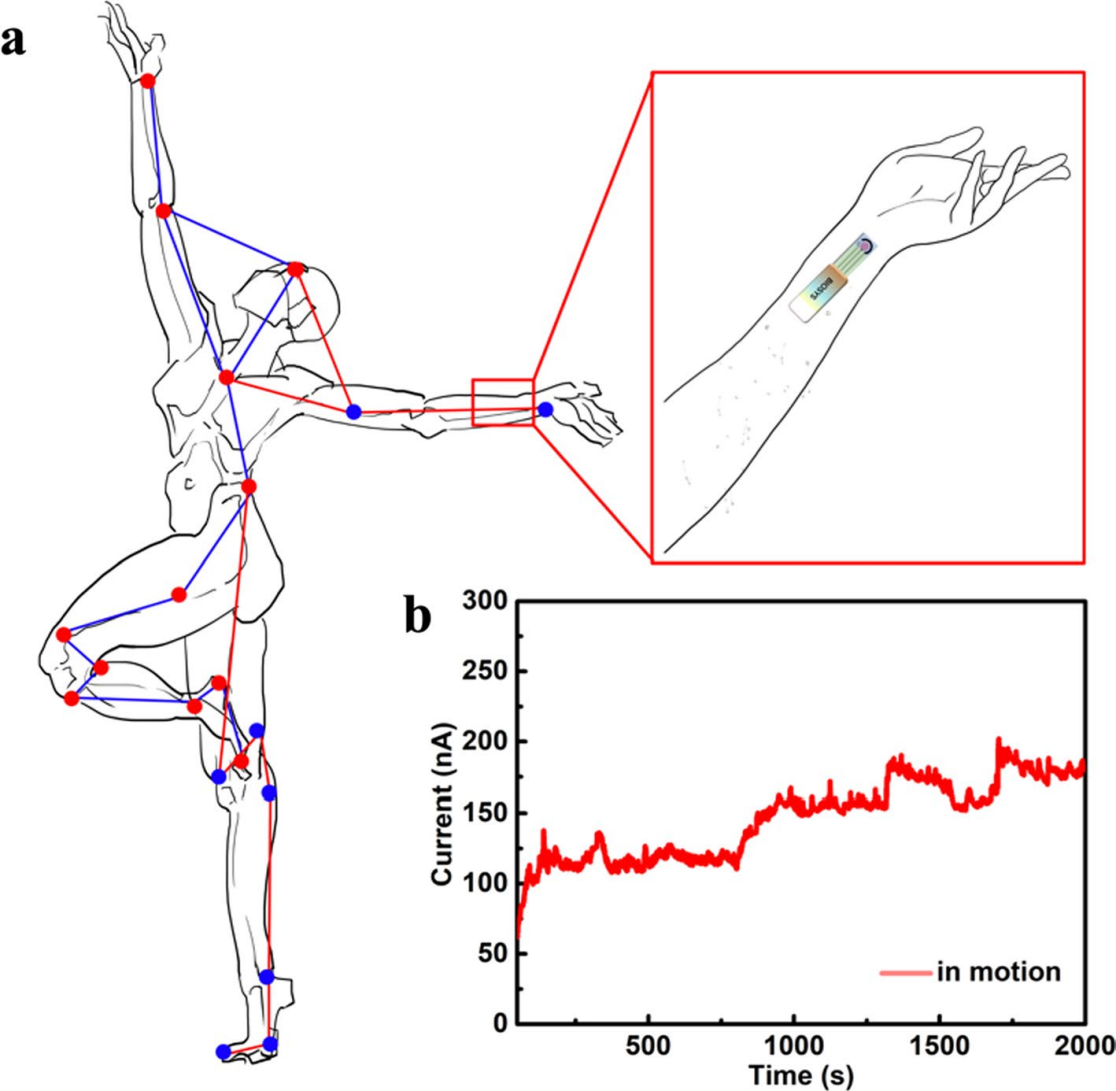
**a** The schematic diagram of dancer in dancing with glucose monitor. **b** The response signal of the volunteer in (**a**)

In order to evaluate the practical application of the prepared wearable biosensor, the concentration of glucose in human sweat during dancing processes was detected using NiMn-MOF electrochemical detector. As shown in Fig. 7a, the response signals of glucose in human sweat were recorded by the BIOSYS intelligent biosensing system in hands of the dancer. Figure 7b reveals the response current curve of NiMn-MOF monitor to glucose in sweat during the exercise. With the increase of exercise time, the response current of the monitoring biosensor to glucose in sweat gradually increased from 64 to 183 nA. It has been reported that, when the volunteers exercised, the concentration of glucose in the sweat increased with continuous sweating, so that the response current increased significantly as the sensor was exposed to the skin [4]. The accurate results of NiMn-MOF wearable sensor further demonstrate the possible application in real time glucose health monitoring.

## Conclusion

In summary, ultrathin two-dimensional nanosheets were synthesized by simple hydrothermal method. The prepared NiMn-MOF possesses excellent electrochemical performance to glucose detection, which is attributed to the introduction of heterogeneous metals and the adjustment of ultra-thin sheets morphology. The sensitivity of NiMn-MOF are 1576 (0–0.205 mM), 1285 (0.255–2.655 mM) and 755.7 μA mM^−1^ cm^−2^ (3.655–5.655 mM), respectively. In addition, the low limitation of detection about 0.28 μM (*S*/*N* = 3), long-term stability and steady response for glucose in sweat further demonstrated the potential of ultra-thin NiMn-MOF as a wearable biomarker sensing material in sweat glucose detection of dancer.

## Data Availability

Data underlying the results presented in this paper are not publicly available at this time but may be obtained from the authors upon reasonable request.
